# Evaluation of the causal effects of blood metabolites on irritable bowel syndrome: Mendelian randomization

**DOI:** 10.1186/s12876-023-03111-9

**Published:** 2024-01-05

**Authors:** Yu Zeng, Huabing Liu, Zhihui Pei, Rui Li, Zuihui Liu, Chuanwen Liao

**Affiliations:** 1 Jiangxi Medical College, Nanchang University, Nanchang, 330006 China; 2 Gastrointestinal Hernia Surgery, Jiangxi Provincial People’s Hospital, The First Affiliated Hospital of Nanchang Medical College, Nanchang, 330006 China

**Keywords:** Irritable bowel syndrome, Blood metabolites, Mendelian randomization

## Abstract

**Background:**

Irritable bowel syndrome (IBS) is a common functional gastrointestinal disorder characterized by abdominal pain, discomfort, and changes in bowel habits. The mechanism underlying IBS remains unclear, and little evidence exists for clarifying the causal relationship between blood metabolites and IBS.

**Methods:**

We conducted a Mendelian randomization (MR) study using two samples. Exposure data for 7824 Europeans were extracted from a genome-wide association study (GWAS) on metabolite levels. The IBS GWAS data from the GWAS database were used for the initial analysis. The primary analysis of causal relationships was conducted using inverse-variance weighting (IVW) with MR-Egger and weighted medians as supplementary analyses. Sensitivity analyses were performed using a combination of the Cochran’s Q test, MR-Egger intercept test, Mendelian randomization pleiotropy residual sum and outlier, and leave-one-out analysis. For significant associations, replication and meta-analyses were performed using additional independent IBS case GWAS data released by the FinnGen Consortium R9. To identify the metabolites, score regression, confounding analysis, and reverse MR were performed to further assess the causal relationships between the metabolites.

**Results:**

After rigorous screening, we identified four known metabolites to be associated with IBS (stearate, odds ratio [OR]: 0.74, 95% confidence interval [CI]: 0.59–0.92; arginine, OR: 1.36, 95% CI: 1.07–1.74; 1-palmitoylglycerol, OR:1.49, 95% CI: 1.07–2.07; 1-palmitoylglycerophosphoinositol, OR: 0.84, 95% CI: 0.71–0.99).

**Conclusions:**

MR analysis revealed a causal relationship between the four metabolites and IBS, providing preliminary evidence for the pathogenesis of IBS. Our results provide novel insights into the potential biomarkers of IBS.

**Supplementary Information:**

The online version contains supplementary material available at 10.1186/s12876-023-03111-9.

## Background

Irritable bowel syndrome (IBS) is a common functional gastrointestinal illness characterized by abdominal pain, discomfort, and altered bowel patterns [1]. IBS is a symptom cluster brought on by a number of illnesses rather than a single condition. IBS development is influenced by altered metabolites, gut microbiota, intestinal immune function, motility, visceral sensation, brain-gut connections, and psychosocial state [2]. Changes in the gut microbiota are believed to play a role in the pathophysiology of IBS; however, the exact cause is unknown, and the illness is frequently accompanied by mental disorders such as anxiety and depression [3]. Various metabolites play pivotal roles in the pathophysiology of IBS [4]. Metabolites mediate numerous biological functions such as signal transmission and immune system control. Individuals with IBS have lower blood levels of short-chain fatty acids [5]. A more comprehensive and systematic investigation of the interactions between blood metabolites and IBS will enhance our understanding of the disease.

Recently, modern omics technologies, including metabolomics, have actively contributed to exploring the mechanisms underlying diseases [6]. Metabolomics involves the qualitative and quantitative assessment of metabolites (small molecules < 1.5 kDa) in bodily fluids. Owing to the intrinsic sensitivity of metabolomics, detecting subtle alterations in biological pathways is possible, which enhances the understanding of the mechanisms underlying various physiological states and abnormal processes, including diseases [7]. Over the past decade, metabolomics has been increasingly used to identify disease biomarkers, and it is regarded as an extremely powerful tool with immense potential for clinical translation [8]. The metabolome and associated pathways have enhanced our understanding of the pathophysiology and mechanisms underlying various diseases. However, studies on the risk relationship between blood metabolites and IBS are limited.

Mendelian randomization (MR), a causal inference method, has been extensively applied in genetic epidemiology [9]. Traditional observational studies are unable to eliminate the effects of environmental confounders. A definitive causal relationship cannot be established even when a strong statistical association is observed between exposure and outcome [10]. Compared with traditional observational studies, MR, employing genetic variants as instrumental variables (IVs), represents a broadly endorsed approach for mitigating potential confounders [11]. This method effectively circumvents the interference of reverse causation and confounders, thereby enabling a more accurate inference of the causal relationship between the exposure and outcome [12].

Stringent randomized controlled trials are highly recommended to determine causal associations; however, their implementation is often hindered by ethical considerations, temporal constraints, and spatial limitations. In the present study, MR was used to investigate the relationship between 486 blood metabolites and IBS. The primary objective of this study was to clarify the causal connections between blood metabolites and IBS and reveal the underlying mechanisms contributing to its onset.

## Methods

### Study design

**Fig. 1:**
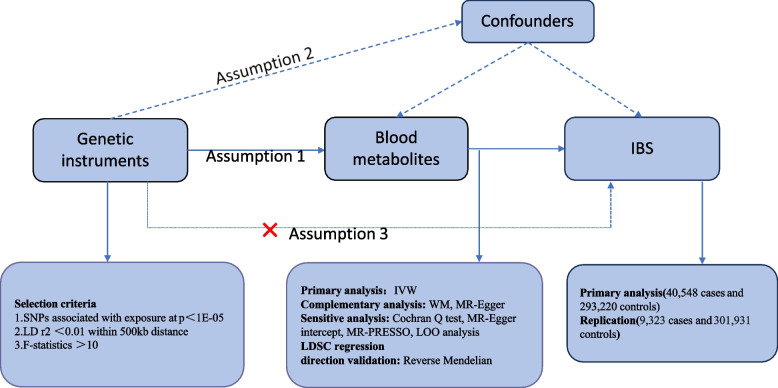
The study design of the correlation between blood metabolites and irritable bowel syndrome. Abbreviations: Assumption 1:the exposures of interest are significantly related to genetic instruments; Assumption 2: genetic tools are independent of confounding variables; Assumption 3: genetic tools are not linked to outcomes and only impact them through exposures

For MR analysis, three assumptions need to be met. These include the association assumption: a robust strong correlation exists between genetic variation (Z) and exposure factor (X); the independence assumption: genetic variation (Z) is independent of the confounding factors (U) influencing the “exposure factor (X) - outcome (Y)” relationship; the exclusion restriction assumption: genetic variation can only influence the outcome through the exposure factors, and cannot affect the outcome via other pathways [13]. This study utilized two sample MR and Mendelian randomization pleiotropy residual sum and outlier (MR-PRESSO) software packages, both implemented in the R software (4.2.3) (Fig. 1).

### Data source

Genetic information on blood metabolites was obtained from the Metabolomics Genome-Wide Association Study (GWAS) server (https://metabolomics.helmholtz-muenchen.de/gwas/). Currently, this is the most thorough documentation available for the genetic loci of blood metabolites, made possible by a genome-wide association scan and advanced metabolic analysis performed by Shin et al. who identified ~ 2.1 million SNPs linked to 486 metabolites associated with human genetic variation [14]. The study included 7824 European citizens, including 6056 participants from the UK Twin Study and 1768 from the German KORA F4 Study. Of the 486 metabolites analyzed, 107 were categorized as unidentified because their chemical properties were ambiguous. Furthermore, 309 metabolites were chemically verified and were distributed across eight major metabolic categories: amino acids, carbohydrates, cofactors, vitamins, energy, lipids, nucleotides, peptides, and exogenous metabolism [15].

The GWAS summary data for IBS was sourced from the GWAS Catalog (https://www.ebi.ac.uk/gwas/) with accession number GCST90016564. This data originates from a large-scale IBS meta-analysis by Eijsbouts and colleagues published in Nature Genetics [16]. The sample includes 40,548 participants of European descent from the UK Biobank, meeting IBS diagnostic criteria based on DHQ Rome III symptom data, self-reported past IBS medical diagnosis, or electronic medical records [16].

### Instrumental variable (IV) selection

Given the limited number of SNPs identified through stringent filtering, we relaxed the significance threshold to *P* < 1 × 10^-5, without altering other selection criteria. Subsequently, we designated linkage disequilibrium (LD) r2 <  0.01 within a 500 KB range, a standard commonly adopted in similar studies [16, 17]. In reverse causation, considering that the GWAS data for IBS possess a sufficient number of significant SNPs, to ensure that the instrumental variables in the model have adequate strength and thereby avoid potential weak instrument bias and violations of the exclusion restriction, we have chosen a genome-wide significance threshold (*P* < 5 × 10^− 8) as the criterion for including SNPs, and LD parameters set to r^2 = 0.001 and kb = 10,000 as conditions for removing LD. A rule of thumb is that the strength of the IVs, as measured by the F-statistic, should be at least 10. When the F-statistic is below 10, the estimates of causal effects can be severely biased [18]. However, a strict threshold may lead to an underestimation of genetic effects by excluding relevant IVs. Using an F-statistic threshold of F = 10 provides a balance, retaining robust instruments while minimizing the risk of weak instrument bias. Therefore, we calculated the R^2^ and F values for each SNP and excluded SNPs with an F value less than 10. Palindromic SNPs with intermediate-effect allele frequencies were excluded. Finally, the preserved SNPs were used for MR analysis.

### Statistical power calculation

The statistical power was calculated using an online tool (https://shiny.cnsgenomic.org/mRnd/) [19, 20]. This tool calculates power using the asymptotic theory to detect causal effects inferred from IVs. The Type I error rate was set at 0.05, and power was computed using the R^2^ of IV, the proportion of cases with outcomes, and odds ratios (OR) obtained through IVW analysis.

### Statistical analysis and sensitivity analysis

Following the above screening criteria, 486 blood metabolites were considered exposure variables. IVW random effects were used to analyze the causal relationship between blood metabolites and IBS. Assuming no horizontal pleiotropy for all SNPs, IVW estimates were derived from a summary study of Wald ratios for all genetic variations. Under this premise, IVW provides the most accurate assessment of causal effects [21]. We used the weighted median (WM) and MR-Egger methods for additional analyses to strengthen the dependability of the results and ensure their accuracy [22]. Four analytical methods were used for the sensitivity analysis: MR-PRESSO, MR-Egger intercept, Cochran-Q test, and leave-one-out analysis (LOO). MR-PRESSO is a recently developed MR method designed to detect and correct abnormal horizontal pleiotropy values, thereby providing accurate estimates [23]. The MR-Egger intercept was calculated to assess the horizontal pleiotropy better and reduce the risk of bias from faulty instrumental variables (IVs) and horizontal pleiotropy [24]. Cochran’s Q test was used to assess heterogeneity among SNPs [25]. LOO was performed to evaluate the influence of each variable on causal estimates by sequentially removing one SNP from the analysis to ascertain if a single SNP predominantly influenced the findings [26].

Employing the False Discovery Rate (FDR) method, the *p*-values of the IVW analysis results for 486 metabolites were corrected. Metabolites with an FDR less than 0.05 were considered to have a relatively convincing causal relationship [27]. In contrast, metabolites with an FDR greater than 0.05 but a p-value less than 0.05 were deemed to have a nominally significant causal relationship and were identified as potential risk factors.

### Replication and meta-analysis

Despite setting strict screening criteria and conducting a sensitivity analysis to ensure data reliability, we repeated the IVW analysis in another IBS cohort using GWAS data of IBS cases released by the FinnGen consortium R9 (https://r9.finngen.fi/). Specifically, these data were obtained from a meta-analysis of GWAS conducted on 9323 Finnish patients with IBS and 301,931 controls. For this investigation, we utilized GWAS data with the accession number GCST012879, and for the replication, we used GWAS data from the FinnGen collaboration. Through a meta-analysis of the two MR analyses, we ultimately determined the blood metabolites that were responsible for the causative effects on IBS. The Review Manager (version 5.3) random-effects IVW model was used to perform the meta-analysis. This experiment was performed according to the protocol described by Cai et al. [28].

### Analysis of genetic correlation

Genetic correlation is a key population parameter that describes the shared genetic architecture of complex traits and diseases [29]. Due to the genetic correlation between traits, MR estimates might violate causal effects [30]. To ensure that the causal effects are not confounded by the genetics of exposure and outcome, we used linkage disequilibrium score regression (LDSC) to examine the genetic correlation between selected metabolites and IBS.

### Analysis of confounding and reverse causation

Despite conducting an array of sensitivity analyses to assess the horizontal pleiotropy of the MR results and identify any SNPs that contravened the MR assumptions, some residual confounding SNPs may persist. We examined the metabolites in the IVs using the Phenoscanner V2 website (http://www.phenoscanner.medschl.cam.ac) to evaluate the association of each SNP with known IBS risk factors, including psychological factors, such as anxiety [31]. To explore whether IBS has any causal effect on the identified important blood metabolites, we used IBS-related SNPs as IVs for reverse MR analysis (i.e., IBS as the exposure and identified blood metabolites as the outcome for MR analysis) [10].

## Results

**Fig. 2:**
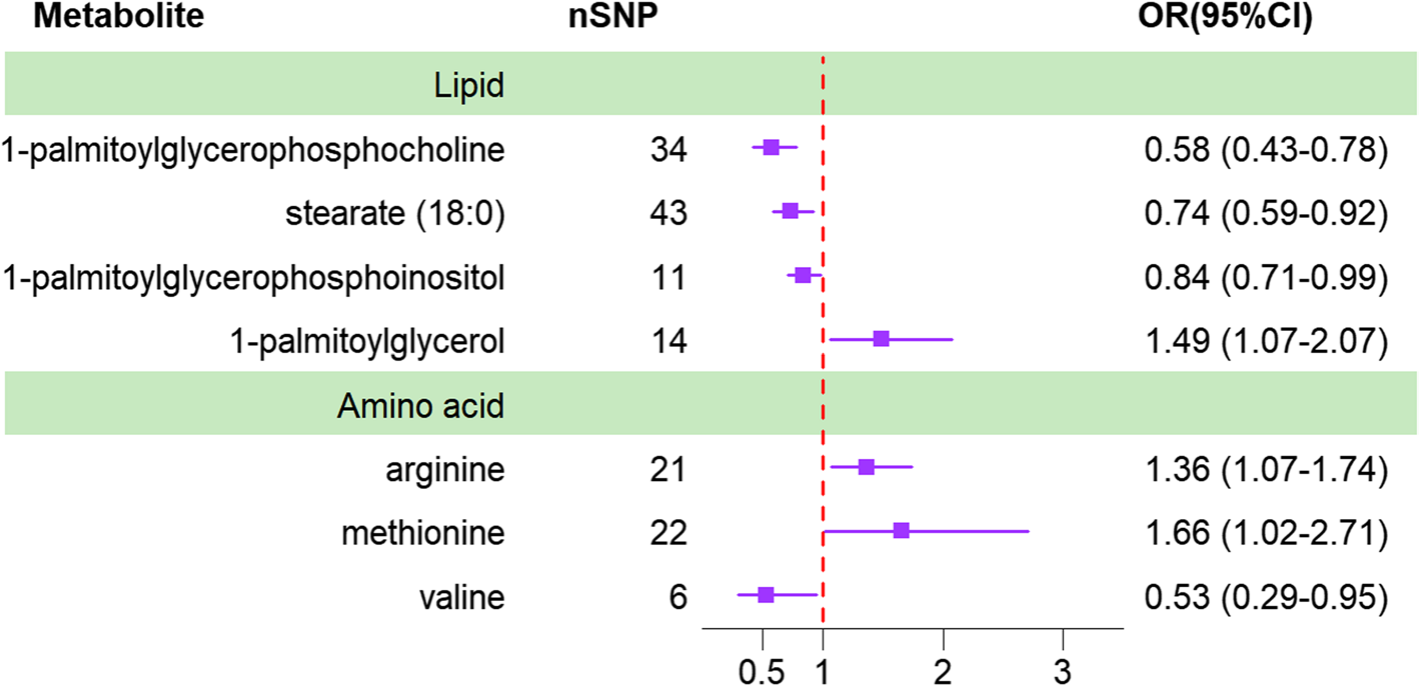
Forest Plot of Causal Associations Between Blood Metabolites and IBS Based on European Ancestry Database. IBS: irritable bowel syndrome; IVW: inverse variance weighting

**Fig. 3:**
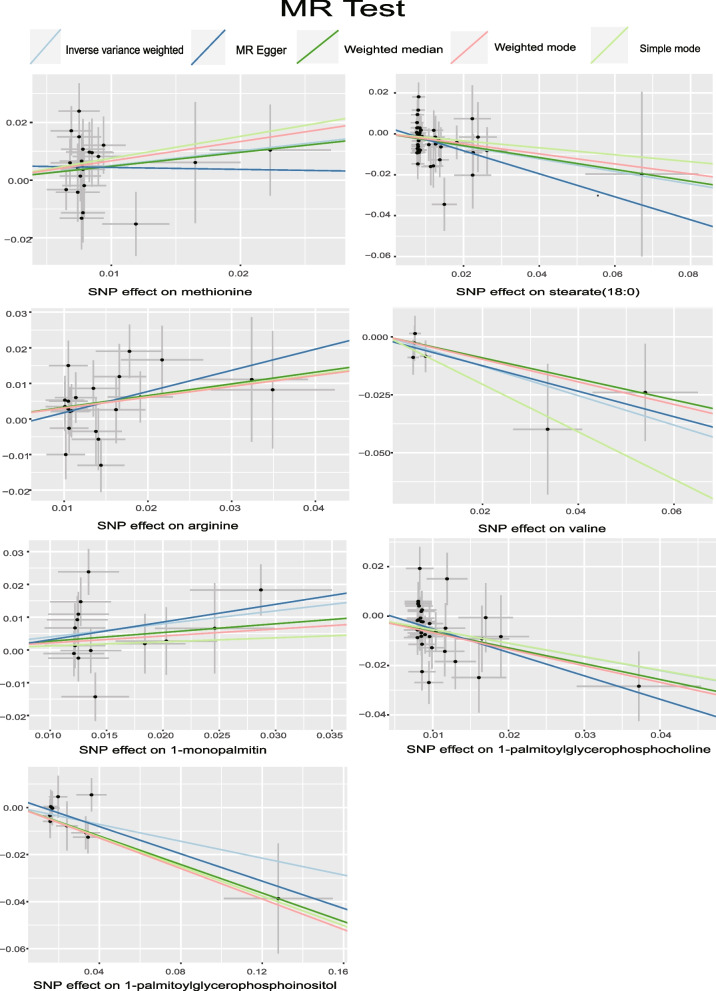
Scatterplot showing the statistically significant MR relationship between metabolites and IBS (*P* < 0.05). IBS: irritable bowel syndrome; MR: Mendelian randomization

Utilizing the IVW method as the primary evaluative approach, we identified 34 metabolites exhibiting a nominally significant causal association with IBS (*P*< 0.05). This cohort comprises 14 metabolites whose identities are currently unknown, in addition to 20 known metabolites. However, it is regrettable to note that no metabolites were found to retain significant association following correction for the FDR (*P*< 0.05). These mainly include lipids, such as 1-palmitoyl glycerophosphocholine; amino acids, such as serotonin; polypeptides; and sugars. Upon further analysis, and following the integration of complementary and sensitivity analyses, we identified seven metabolites that conformed to stringent selection criteria, thereby establishing them as candidate substances. Overall, the direction and scale of the estimates for these seven metabolites remained consistent across the three analytical methods (IVW, MR-Egger, and WM), and the IVW estimates were significant (*P* < 0.05). Additionally, the MR-PRESSO (*P* > 0.05), Cochran’s Q test (*P* > 0.05), and MR-Egger intercept test (*P* > 0.05) indicated a lack of heterogeneity and pleiotropy. The LOO analysis outcomes reinforced the idea that the MR estimation was not biased by a single SNP. Therefore, these seven blood metabolites were selected for subsequent analysis (Figs. 2 and 3, Table 1).

**Table 1:** Three MR models tested for heterogeneity and horizontal pleiotropy and assessed the causal associations between seven recognized metabolites and the probability of IBS

Metabolite	Methods	SNP (*n*)	OR (95%CI)	P	Heterogeneity		Pleiotropy Intercept		Power
					Q	P	P		
1-palmitoylglycerophosphocholine	IVW	34	0.58 (0.43–0.78)	0.0003	37.82	0.26	0.004	0.35	0.99
	WM	34	0.53 (0.35–0.80)	0.002					
	MR Egge	34	0.39 (0.16–0.93)	0.043					
stearate (18:0)	IVW	43	0.74 (0.59–0.92)	0.007	38.39	0.63	0.003	0.35	0.88
	WM	43	0.75 (0.55–1.02)	0.069					
	MR Egger	43	0.57 (0.32–1.02)	0.063					
1-palmitoylglycerophosphoinositol	IVW	11	0.84 (0.71–0.99)	0.034	6.25	0.79	0.003	0.45	0.97
	WM	11	0.74 (0.59–0.92)	0.008					
	MR Egger	11	0.75 (0.53–1.05)	0.131					
1-palmitoylglycerol	IVW	14	1.49 (1.07–2.07)	0.019	21.27	0.067	0.002	0.81	0.84
	WM	14	1.30 (0.89–1.91)	0.170					
	MR Egge	14	1.72 (0.54–5.48)	0.380					
arginine	IVW	21	1.36 (1.07–1.74)	0.012	20.21	0.45	−0.004	0.46	0.86
	WM	21	1.39 (0.99–1.95)	0.057					
	MR Egger	21	1.81 (0.83–3.95)	0.152					
methionine	IVW	22	1.66 (1.02–2.71)	0.04	21	0.52	0.005	0.51	1.00
	WM	22	1.62 (0.80–3.26)	0.057					
	MR Egger	22	0.93 (0.16–5.45)	0.152					
valine	IVW	6	053 (0.29–0.95)	0.03	5	0.86	−0.001	0.77	0.82
	WM	6	0.63 (0.30–1.32)	0.22					
	MR Egger	6	0.58 (0.26–1.29)	0.25					

*OR* odds ratio, *SNP* single-nucleotide polymorphisms, *IVW* inverse variance weighting, *MR* Mendelian randomization, *WM* weighted median

### Replication and meta-analysis

**Fig. 4:**
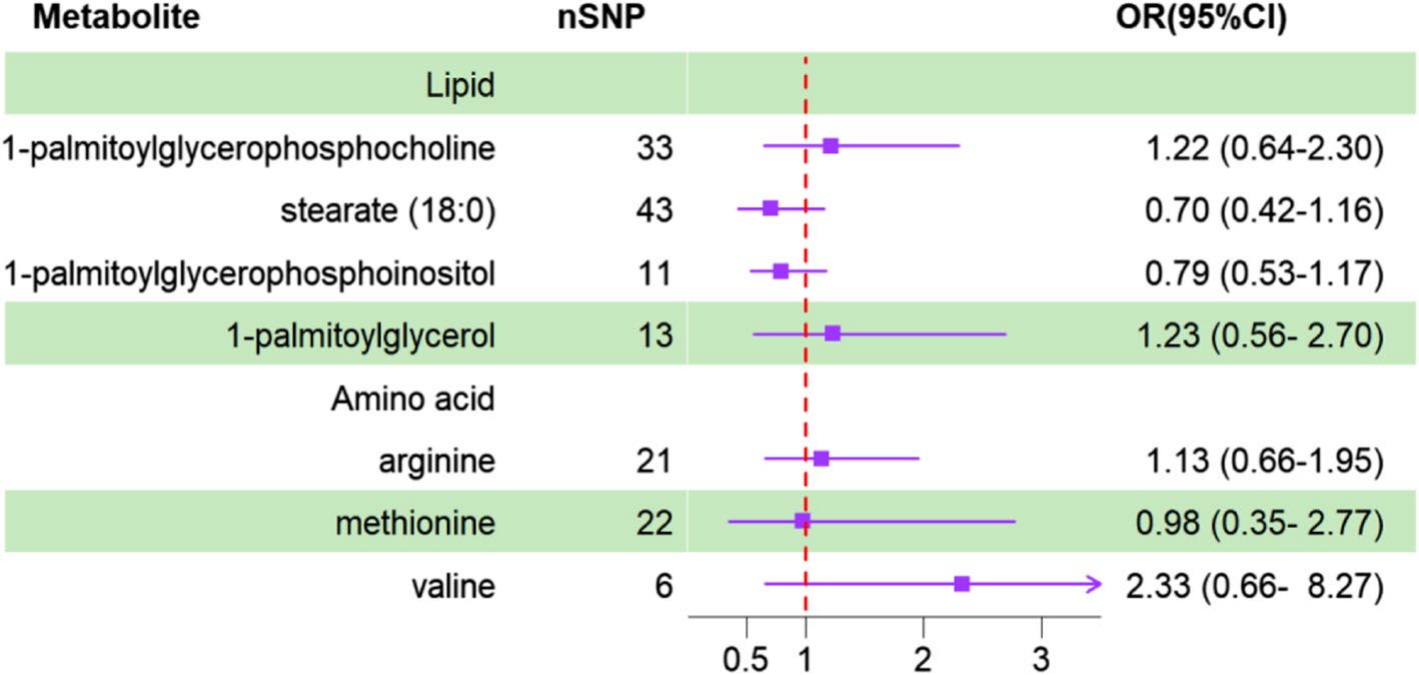
Forest Plot of Causal Associations Between Blood Metabolites and IBS Based on Finnish Database

**Fig. 5:**
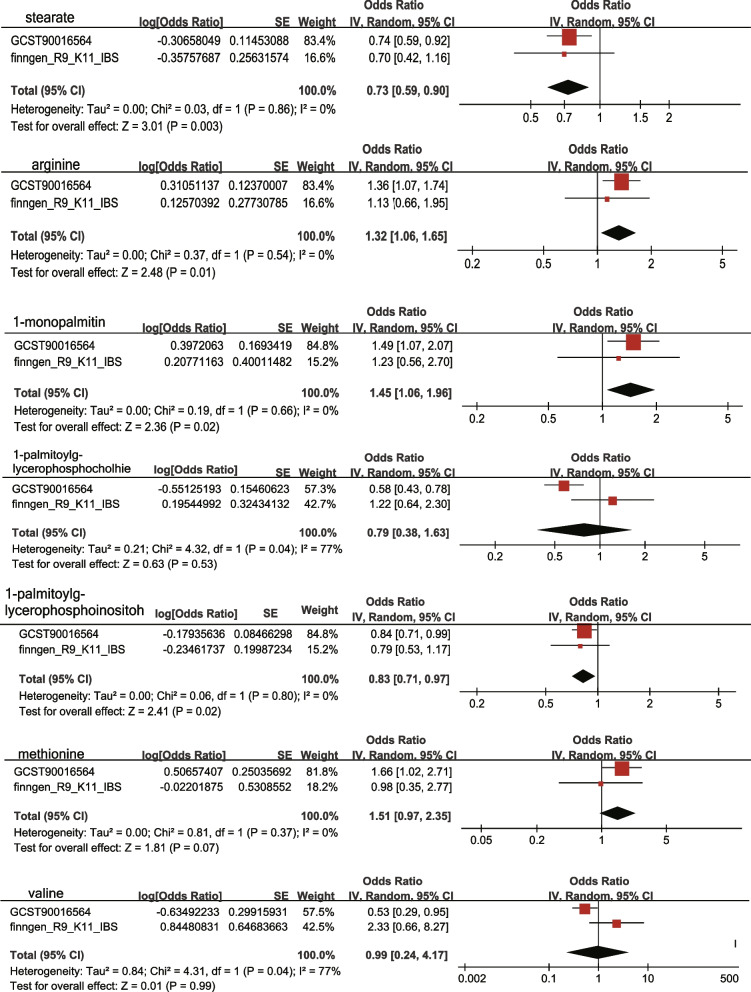
Meta-analysis of the relationships between IBS and metabolites. IBS: irritable bowel syndrome

We used GWAS data for IBS from another cohort downloaded from the FinnGen Alliance R9 and repeated the above MR analysis. We used GWAS data for IBS from another cohort downloaded from FinnGen Alliance R9 and replicated the above-mentioned MR analyses. We found that the replication results for Methionine, 1-palmitoylglycerophosphocholine, and Valine showed considerable differences, while Stearate, Arginine, 1-palmitoylglycerol, and 1-palmitoylglycerophosphoinositol yielded similar results, although they did not reach statistical significance. Meta-analysis was performed using random effects in Review Manager software (version 5.3, [32]. Our results showed similar outcomes. A previous meta-analysis has identified four metabolites associated with IBS. Methionine, 1-palmitoylglycerophosphocholine, and valine were excluded because of insignificant results (Figs. 4 and 5).

### Analysis of genetic correlation

According to the results of the genetic correlation analysis, evidence of a genetic correlation between stearate and others in IBS is weak. This indicates that the shared genetic components did not confound the MR results (Table 2).

**Table 2:** The genetic correlations between Metabolites and IBS

Traits1	Trait2	Rg	Rg-se	Rg-p
Stearate (18:0)	IBS	0.097	0.057	0.089
Arginine	IBS	−0.549	0.338	0.105
1-palmitoylglycerol (1-monopalmitin)	IBS	−0.055	0.069	0.424
1-palmitoylglycerophosphoinositol	IBS	−0.077	0.117	0.508

*Rg* genetic correlation

### Analysis of confounding and reverse causation

**Fig. 6:**
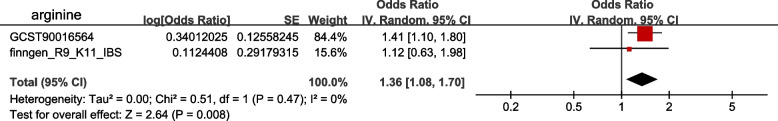
Meta-analysis of causality between arginine and irritable bowel syndrome after removal of confounding SNPs. SNP: single nucleotide polymorphism

For each SNP of the identified 20 metabolites, we searched for phenotypes associated with exposure and the resulting variables. In the IVs of arginine and N-acetyl aspartic acid, we found that rs837763 and rs11030392 seemed to be related to mental state, but this did not affect our results. After excluding these confounding IVs, the results of arginine were still significant (odds ratio [OR]:1.41, 95% confidence interval [CI]:1.10–1.80), and the final meta-analysis results were significant (OR:1.36, 95% CI:1.08–1.70). In the reverse MR analysis, none of the 20 known metabolites showed a reverse causal relationship with IBS. Only the unknown metabolites X-11438, X-11521, X-14374, and X-13741 may have a reverse causal relationship with IBS (Fig. 6).

## Discussion

IBS has a complex pathophysiology affected by a wide range of variables, including changes in the gut-brain axis, visceral hypersensitivity, abnormalities in intestinal secretion, and intestinal permeability [33]. IBS, as a common gastrointestinal functional disorder, presents significant challenges due to the lack of objective diagnostic methods. Currently, it remains a diagnosis based on symptoms, and there is a notable absence of sensitive biological markers. At present, the international common is the Rome IV diagnostic criteria: Recurrent abdominal pain or discomfort occurring at least 3 days per week over the last 3 months, with symptom onset at least 6 months prior to diagnosis, and associated with two or more of the following conditions: (1) Related to defecation, (2) Associated with a change in the frequency of stool, (3) Associated with a change in the form (appearance) of stool [34]. The quality of life of IBS patients is often severely impacted by pain, functional disturbances, and accompanying psychological conditions such as depression and anxiety [35].

Various metabolites can act as signaling molecules that affect biological processes, and changes in host metabolites may be associated with the emergence of IBS symptoms [36]. IBS is exacerbated and maintained by changes and variations in intestinal metabolites. Han et al. highlighted the significant alterations in serum metabolites that aid in the diagnosis of IBS patients, proposed a potential role for metabolic dysregulation in its pathophysiology, and offered fresh information on the co-occurrence of IBS and depression [4]. In our study, we endeavor to provide new insights into the causal role of blood metabolites in the risk of IBS. This is pivotal as it suggests that screening for certain blood metabolites could be a viable strategy for identifying individuals at high risk for IBS. Future research could focus on validating these metabolites as potential biomarkers for IBS, which would significantly advance our understanding and management of this condition.

The current study is the first in-depth MR investigation to examine the connection between IBS and human blood metabolites. For the first time, our study used rigorous MR methods to set strict screening criteria. It verified the GWAS data of large IBS cohorts in two different queues, ultimately determining a causal relationship between the four metabolites and IBS and providing potential inspiration for further precision treatments. These molecules included three lipids and one amino acid. The lipids include stearate, 1-monopalmitin, and 1-palmitoylglycerophosphoinositol, and the amino acid was arginine. Among these, 1-monopalmitin and arginine may potentially be associated with an increased risk for IBS, while stearate and 1-palmitoylglycerophosphoinositol appear to be potential protective factors against IBS.

The role of bioactive lipids in the gut-brain axis and the ability of palmitoylglycolamide to lower inflammatory markers in mouse models of inflammatory bowel disease (IBD) and butyrate produced by the gut microbiota to effectively reduce inflammation and pain in animal models of IBS and IBD have been demonstrated in studies by Russo [37]. Numerous animal and plant fats contain stearate, an 18-carbon saturated fatty acid. Stearate is crucial for the growth of different tumors because it alters mitochondrial morphology, rendering cancer cells vulnerable to oxidative stress and cell death [38]. Compared with other saturated fatty acids, stearate is associated with reduced low-density lipoprotein cholesterol, and low low-density lipoprotein (LDL) levels seem to be related to the manifestation of IBS symptoms [39, 40]. Additionally, the stearate content is significantly decreased in diarrhea caused by chemotherapy [41]. Stearates are also associated with intestinal permeability, which may explain their causal association with IBS. Current evidence indicates that stearate, a saturated fatty acid, serves as an agonist of Toll-like receptor 4, initiating an inflammatory response and leading to alterations in the gut microbiota [42].

Recent research has shown that lipid metabolism disorder is present in IBS and is an important pathophysiological characteristic of this disease [43]. Although the roles of 1-monopalmitin and 1-palmitoylglycerophosphoinositol in IBS have not yet been reported, studies have verified their potential to regulate the immune system [44]. This could be a promising direction for further investigation.

The urea cycle and metabolism of arginine and proline include the semi-essential amino acid arginine. In humans, arginine metabolism mainly produces urea and ornithine via the arginase pathway and nitric oxide (NO) and citrulline via the nitric oxide synthase (NOS) pathway [45]. In the digestive system, NO serves as the primary inhibitory nonadrenergic and noncholinergic neurotransmitter [46]. Gastrointestinal motility is directly regulated by inhibitory and excitatory motor neurons in the smooth muscle layer. NO released from nerve stimulation in the myenteric plexus causes smooth muscle relaxation and plays a critical role in regulating esophageal, gastric, and intestinal peristalsis [47]. NO may play a role in the pathogenesis of visceral hypersensitivity in IBS, according to Kuiken et al. [48]. Higher blood levels of l-arginine and endogenous methylarginines have also been linked to adult IBS, according to the available research [49].

The MR analysis employed in this study had several advantages. This is the most exhaustive and methodological probe for determining a causal relationship between blood metabolites and IBS. Second, we adopted rigorous criteria to circumvent inevitable pitfalls encountered in previous studies, such as reverse causation and confounding biases. Specifically, several strategies were implemented to ensure the eradication of elements that contravened the MR assumptions, thereby engendering reliable results. The uniformity across the three MR estimation directions and sensitivity analyses indicated the robustness of the findings. Furthermore, the veracity of the results was corroborated by the replication and meta-analysis of GWAS data from a separate IBS cohort. Fourth, we evaluated the genetic association between metabolites and IBS using LDSC, which made the MR Estimate more convincing.

There are certain limitations to the present study. First, few SNPs can be identified at the whole-genome level. To address this issue, we implemented a slightly loosened threshold for the MR analysis, a strategy frequently used in previous studies. Second, most of the metabolite data was of European populations, which restricts the applicability of our findings to other racial groups. Meanwhile, we conducted a meta-analysis combining the European and FinnGen cohorts, yet the ancestral differences between Finland and other parts of Europe might introduce some heterogeneity in the association effects between these cohorts, impacting the reliability of the results. Third, although a relatively wide range of metabolites was included in this study, several of their roles and disease-related processes remain unknown, making it difficult to interpret the results of the MR analysis. Finally, although this study identified several metabolites linked to IBS, further investigations are needed to clarify their functions in the pathogenesis of the disease.

## Conclusions

In this study, we determined a causal relationship between the four metabolites and IBS through MR analysis, providing preliminary evidence of the pathogenesis of IBS. Our results provide novel insights into the potential biomarkers of IBS. However, further studies are required to validate these findings.

### Supplementary Information

**Additional file 1.**

## Data Availability

The datasets used in this study are accessible through online repositories. The article/Supplementary Material includes repository name(s) and accession number(s). Genetic association estimates for IBS were sourced from published GWAS and the FinnGen database. We are deeply appreciative of all participants and researchers who shared these valuable datasets.

## Abbreviations

GWAS: Genome-wide association study
IBD: Inflammatory bowel disease
IV: Instrumental variable
IVW: Inverse variance weighting
IBS: Irritable Bowel Syndrome
FDR: False Discovery Rate
LOO: Leave-one-out analysis
LD: Linkage disequilibrium
LDSC: Linkage disequilibrium score regression
MR: Mendelian randomization
LDL: Low-density lipoprotein
NOS: Nitric oxide synthase
WM: Weighted median
